# Pasta Incorporating Olive Pomace: Impact on Nutritional Composition and Consumer Acceptance of a Prototype

**DOI:** 10.3390/foods13182933

**Published:** 2024-09-16

**Authors:** Diana Melo Ferreira, Bárbara C. C. Oliveira, Carla Barbosa, Anabela S. G. Costa, Maria Antónia Nunes, Maria Beatriz P. P. Oliveira, Rita C. Alves

**Affiliations:** 1 LAQV/REQUIMTE, Department of Chemical Sciences, Faculty of Pharmacy, University of Porto, Street of Jorge Viterbo Ferreira, 4050-313 Porto, Portugal; dimelo@ff.up.pt (D.M.F.); barbaraoliveira1998@gmail.com (B.C.C.O.); cbarbosa@estg.ipvc.pt (C.B.); acosta@ff.up.pt (A.S.G.C.); antonianunes.maria@gmail.com (M.A.N.); rcalves@ff.up.pt (R.C.A.); 2 CISAS/IPVC, Polytechnic Institute of Viana do Castelo, Avenue of Atlantic, 4900-348 Viana do Castelo, Portugal

**Keywords:** agri-food by-products, food industry, food security, sustainability, macronutrients, vitamin E, fatty acids, phenolic compounds, antioxidant activity, sensory analysis

## Abstract

The food industry is encouraged to develop new sustainable foodstuffs, and agri-food by-products can serve as valuable ingredients in these formulations. In this work, olive pomace (OP), a by-product of olive oil production, was incorporated as an ingredient in pasta. The changes in the nutritional composition and consumer acceptance were assessed, aiming to scale up the production. OP contains dietary fibre (55%), fat (9%), α-tocopherol (43 mg/kg), and oleic acid (76%) after moisture elimination. For that, the following two drying procedures were tested: 40 °C for 48 h (OP40) and 70 °C for 24 h (OP70). Both samples were sieved to remove the stone pieces. Drying at 70 °C (OP70) was the fastest method, revealed a better nutritional profile than OP40, and was the product selected for the incorporation into the pasta. The enriched pasta, containing 7.5% of OP70, was compared to a control. It showed an improved nutritional value with higher contents of fat, ash, fibre, vitamin E, oleic acid, phenolics, and flavonoids, a composition related to potential health benefits. Consumers appreciated the appearance, colour, shine, and aroma of the obtained pasta, making it a prototype with commercial viability. However, several improvements need to be implemented, namely, at the textural levels. Corrective actions, such as the optimisation of the amount of incorporated OP, the use of other ingredients for flavour masking, and textural adjustments, are advisable, thereby making this product more appealing and accepted by a larger number of consumers. This prototype can be a good approach for the circular economy, environmental sustainability, and food security.

## 1. Introduction

The United Nations predict that the world’s population will reach 11 billion people by the end of the century, which will create a shortage of essential resources (e.g., cereals like wheat). Agriculture is also facing significant threats due to climatic change. Hence, the food industry is confronted with a huge challenge—providing healthy sustainable food for the growing population while preventing environmental exhaustion [1].

For those reasons, it is necessary to valorise agri-food by-products that can be raw materials for food production. The majority are discarded or used as animal feed, but, if carefully collected, can be transformed into edible ingredients for food. A key example is the olive oil sector [2,3,4].

Global olive oil production is increasing, reaching 2 M tonnes per year. Mediterranean basin countries alone are responsible for 97% of the world’s production. This production has many health and economic benefits but has a negative environmental impact. As a result of seasonable production, large quantities of by-products are generated in a short period, which requires specific storage conditions for chain operators [5].

Olive pomace (OP), the major by-product, is composed of olive pulp, skin, and stone. For every 100 kg of olives, around 70 to 80 kg of OP are produced. OP is phytotoxic due to its high organic load and richness in bioactive compounds, such as phenolic compounds. Thus, its management can be seen as an economic burden, but, due to its rich composition, it can be a low-cost and valuable ingredient for several applications. For instance, OP can be used to formulate functional foods, but its bitter taste can lead to consumers’ rejection, which is a big drawback. Nonetheless, its valorisation is crucial to achieving olive oil chain sustainability [2,3,4,6,7,8,9,10,11].

OP contains several phenolic compounds that are valuable for food applications, including hydroxytyrosol and its derivatives, tyrosol, oleoside 11-methyl ester, ligstroside aglycone, hydroxyoleuropein, and oleuropein aglycone, all of which belong to the tyrosol subclass. In addition, OP contains flavonoids such as luteolin-7-O-glucoside, apigenin-7-O-glucoside, and luteolin. OP also features verbascoside, a hydroxycinnamic acid, and quinic acid, a cyclitol [4]. These compounds are well-known for their potent antioxidant and anti-inflammatory properties, offering potential health benefits such as reducing oxidative stress and inflammation, supporting cardiovascular health, and potentially providing neuroprotective effects [12]. Our research group has previously demonstrated the anti-microbial [3] and antitumoral activities [11] of OP.

However, OP can also contain contaminants such as mycotoxins (e.g., ochratoxin A), pesticide residues (e.g., chlorpyrifos), and polycyclic aromatic hydrocarbons (e.g., benzo[a]pyrene). These substances may arise from agricultural practices or processing methods and pose risks to food safety and human health if not properly managed. Therefore, rigorous quality control in the processing of OP is critical to ensuring its safe application in food industries [13,14]. Nonetheless, the European Food Safety Authority (EFSA) has reported that pesticide residue levels on olives are among the lowest of all foods available to consumers in Europe [15].

Moreover, the increasing demand for nutritive and healthy foods and the nutritional quality attributes of OP has motivated several researchers to try and develop innovative food products, e.g., enriched/fortified pasta [16,17,18,19,20,21], bakery products [17,18,20,22,23,24,25,26,27,28,29], and fish-based products [30,31]. 

A study used 7% dried OP to fortify Tagliatelle pasta, which retained 6.6 mg/100 mg of hydroxytyrosol and maintained a good resistance and texture after cooking [21]. Another study also found that 10% dried OP in spaghetti improved the phenolic content and antioxidant activity. The incorporation affected the sensory characteristics of uncooked (colour and overall quality) and cooked (colour, taste, elasticity, firmness, bulkiness, and overall quality) spaghetti [20].

Currently, consumers are better informed, and their food choices target not only health and sustainability concerns, but also convenience and innovation. They prefer products classified as functional, organic, sustainable, and natural [32,33]. Therefore, the development of new food products is very challenging. Chemical and sensory analysis are important tools for their characterisation [34]. Particularly, sensory analysis, a scientific method which uses human senses (sight, smell, touch, taste, and hearing), provides unique information about food quality perception [35].

This study aimed to formulate a prototype pasta enriched with OP to add value to this by-product and meet current food demands, ultimately aiming for industrial-scale production. The study involved developing a procedure to dry OP and remove the stone, formulating both control and enriched pasta, and conducting comprehensive chemical analyses (including proximate analysis, lipid fraction analysis, and antioxidant screening) of OP and pasta (uncooked and cooked) samples. Additionally, sensory analysis focusing on consumer acceptability and a preference test were performed on the final pasta samples.

While Balli et al. [21] conducted a more specific evaluation of phenolic compounds using HPLC techniques in OP-enriched pasta, the present study provides a broader nutritional perspective, particularly the proximate composition, energy values, vitamin E and fatty acid profiles, total phenolics and flavonoids, and antioxidant activity in OP-enriched pasta. Overall, we believe that both studies complement each other effectively.

## 2. Materials and Methods

### 2.1. Olive Pomace Samples

The OP was supplied by a local producer (M. C. Rabaçal & Aragão, Lda., Alfândega da Fé, Portugal). It was divided into three batches which were subjected to different preparations, resulting in three different samples. One-third was freeze-dried (Telstar Cryodos-80 Terrassa, Barcelona, Spain) and milled (Thermomix TM5, Vorwerk, Wuppertal, Germany)—the FOP (freeze-dried olive pomace) sample. Another third was dried in an oven (Digitronic-TFT series, J.P. SELECTA, Barcelona, Spain) at 40 °C for 48 h, and then milled and sieved (using stainless-steel sieves with a particle size of 1.4, 1.25, 1, and 0.5 mm) to remove the stone—the OP40 (olive pomace dried at 40 °C) sample. The last third was dried at 70 °C for 24 h, and milled and sieved using the same equipment previously mentioned—the OP70 (olive pomace dried at 70 °C) sample. The OP70 sample was chosen for the pasta enrichment, as will be explained in Section 3.1: “Chemical Analysis of Olive Pomace Samples”.

### 2.2. Pasta Samples

Six pasta formulations with different compositions were produced in the lab, as shown in Table 1. The pasta formulations were manually structured into laces and dried at 50 °C for 5 h to increase the shelf-life [36]. After that, the pasta formulations were cooked in boiling water (Thermomix TM5, Vorwerk, Wuppertal, Germany) at 100 °C for 10 min. Formulations 2 and 6 were chosen for the analysis, as will be explained in Section 3.2: “Chemical Analysis of Pasta Samples”. As a result, four samples were freeze-dried and milled until further analysis—the uncooked control pasta (UCP), cooked control pasta (CCP), uncooked olive pomace dried at 70 °C pasta (UOP70P), and cooked olive pomace dried at 70 °C pasta (COP70P).

### 2.3. Chemicals and Reagents

Merck (Darmstadt, Germany), Sigma-Aldrich (St. Louis, MO, USA), or VWR Chemicals (Alfragide, Portugal) provided all of the chemicals and reagents of analytical or HPLC grade unless otherwise stated. Ultrapure water was attained in a Milli-Q water purification system (Millipore, Bedford, MA, USA).

### 2.4. Chemical Analysis

#### 2.4.1. Proximate Analysis

Proximate analysis was determined according to AOAC procedures [37], particularly, the contents of total fat (Soxhlet method n.° 991.36), ash (incineration method n.° 920.153), total dietary fibre and insoluble fibre (enzymatic–gravimetric method n.° 985.29), and total protein (Kjeldahl method n.° 978.04). The used nitrogen conversion factor was 6.25, and the soluble fibre and available carbohydrate contents were obtained by the difference [38]. The moisture content was determined in an infrared balance (Scaltec model SMO01, Scaltec Instruments, Heiligenstadt, Germany). The results are expressed in g/100 g of sample in dry weight (DW) and fresh weight (FW). Energy values were calculated according to the following equations [39]:

Energy (kJ/100 g) = (g fat × 37) + (g fibre × 8) + (g protein × 17) + (g carbohydrates × 17)(1)

Energy (kcal/100 g) = (g fat × 9) + (g fibre × 2) + (g protein × 4) + (g carbohydrates × 4)(2)

#### 2.4.2. Lipid Fraction Analysis

The lipid fractions of the samples were extracted according to Alves et al. [40], with slight modifications described by Melo et al. [41]. In brief, the solution was homogenised in a Heidolph Multi Reax Vibrating Shaker (VWR International, Radnor, Pennsylvania, USA), tocol was used as the internal standard (0.1 mg/mL), and *n*-hexane was used as the extracting solvent. The resulting extracts were used for the analysis.

Vitamin E profiles were determined in an HPLC system (Jasco, Tokyo, Japan) equipped with a multiwavelength diode array detector (DAD, MD-2015—for identification), a fluorescence detector (FLD, FP-2020—for quantification), and a normal-phase column (75 mm × 3.0 mm, 3.0 μm, Supelcosil^TM^ LC-SI, Supelco, Bellefonte, USA—for separation) using the same conditions previously described by Melo et al. [41]. α-, β-, γ-, and δ-Tocopherols and α-, β-, γ-, and δ-tocotrienols were identified based on their UV spectra and by comparing the retention times with individual standards (Calbiochem, La Jolla, CA, USA). Results are expressed as mg/kg of sample in DW or FW.

Fatty acids (FAs) were derivatised into fatty acid methyl esters (FAMEs) by an acid transesterification reaction according to ISO 12966-2017 [42]. FA profiles were determined in a gas chromatograph (GC-2010 Plus, Shimadzu, Tokyo, Japan) equipped with an automatic sampler, a split/splitless auto-injector (AOC-20i), a flame ionisation detector (FID), and a silica capillary column (50 m × 0.25 mm, 0.20 μm, Agilent J&W CP-Sil 88, Agilent Technologies, California, USA—for separation) using the same conditions previously described by Melo et al. [41]. The FAMEs were identified by comparing the retention times with a standard mixture (FAME 37, Supelco, Bellefonte, PA, USA). Results are expressed in g/100 g of sample in DW (for OP samples), in mg/100 g of sample in DW (for pasta samples), and in relative percentage (%) of total FAs (for all samples). The sums of the saturated fatty acids (SFAs), monounsaturated fatty acids (MUFAs), and polyunsaturated fatty acids (PUFAs) were calculated. The n6/n3 and PUFA/SFA ratios were also calculated.

#### 2.4.3. Antioxidant Screening

The mass/volume ratio was optimised for all extractions, which were carried out with a 50% ethanol/50% water solution (V/V) for 1 h in agitation (Heidolph Multi Reax Vibrating Shaker, VWR International, Radnor, Pennsylvania, USA). The OP and pasta extracts were obtained with 100 and 250 mg of sample, respectively, in 40 mL of extracting solvent. The resulting extracts were used for the analysis.

The total phenolic compound (TPC), total flavonoid content (TFC), ferric reducing antioxidant power (FRAP), and 2,2-diphenyl-1-picrylhydrazyl radical scavenging ability (DPPH^•^-SA) assays were determined according to Ferreira et al. [43]. The calibration curves were prepared with gallic acid (5–100 mg/mL; R^2^ = 0.999), catechin (0–400 µL/mL; R^2^ = 0.999), ferrous sulphate (25–500 μmol/L; R^2^ = 0.999), and Trolox (5.62–175.34 mg/L; R^2^ = 0.998), respectively. The absorbance was read at 765, 510, 595, and 525 nm, respectively, in a microplate reader (Synergy HT GENS5, BioTek Instruments, Winooski, Vermont, USA). Results are expressed as gallic acid equivalents (GAEs), catechin equivalents (CEs), ferrous sulphate equivalents (FSEs), and Trolox equivalents (TEs), respectively.

### 2.5. Sensory Analysis

Consumer acceptability tests were performed for the cooked samples. The pasta cooking procedure involved boiling 250 mL of water with half a coffee spoon of salt. Each pasta sample was cooked separately for 10 min, after which the water was drained. The pasta was then placed on the corresponding plate and wrapped in aluminium foil to keep it warm before the participants consumed it and completed the provided questionnaire.

A single sensory session was conducted, during which two samples were tested. Cooked pasta samples were submitted to a 71-consumer panel (38 women and 33 men), who were recruited around the university campus. Each participant received two samples in white containers holding approximately 10 g of each cooked pasta identified with a random three-digit number. To register consumer opinion, a questionnaire was used that consisted of four sections.

In the first section, participants were asked to indicate their gender, age, and frequency of pasta consumption. In Section 2, participants were asked to indicate their opinion using a 9-point hedonic scale (ranging from 1 = “Extremely unpleasant” to 9 = “Extremely pleasant”), firstly, concerning visual aspects (appearance, colour, and shine) and aroma, and, lastly, concerning mouthfeel aspects (flavour, texture, and flavour persistence). In Section 3, after tasting, the same was asked for the overall acceptability. The buying intention was also evaluated with a 5-point hedonic scale, ranging from 1 = “Would not buy” to 5 = “Would certainly buy”. In the last section, the preference order was assessed.

### 2.6. Statistical Analysis

Statistical analysis was performed using IBM SPSS v. 28 (IBM Corporation, New York, NY, USA). One-way ANOVA was used to assess significant differences between three or four samples, followed by Tukey’s post hoc test to make pairwise comparisons between means. The Independent Samples T-test was used to assess significant differences between two samples. The level of significance for all hypothesis tests was 95% (*p* ≤ 0.05). Experiments were performed in triplicate (*n* = 3). Results are displayed as mean ± standard deviation (s.d.).

## 3. Results and Discussion

### 3.1. Chemical Analysis of Olive Pomace Samples

The results of the chemical analysis of the OP samples are presented in Table 2. The FOP was found to be rich in total fibre (55%) and mostly insoluble (51%) due to the presence of olive stone and carbohydrates (28%). It also presented a considerable amount of fat (9%), primarily composed by oleic acid (76%) and vitamin E (51 mg/kg), mainly α-tocopherol (42 mg/kg). Significant decreases are observable in the contents of total fibre (FOP: 55% vs. dried samples: 47% DW), insoluble fibre (FOP: 51% vs. dried samples: 42–43% DW), and carbohydrates (FOP: 28% > OP70: 25% > OP40: 23% DW). Conversely, significant increases are observable in the values of total protein (dried samples: 7% vs. FOP: 4% DW), fat (dried samples: 17% vs. FOP: 9% DW), ash (dried samples: 6% vs. FOP: 4% DW), and soluble fibre (dried samples: 5% vs. FOP: 3% DW). These findings can be explained by the presence of the stone in the FOP, which was removed by sieving from the dried samples.

The comparison of the dried OP samples led to the selection of OP70 for incorporation into the pasta, since it was the faster drying method (24 h instead of 48 h), which saves electricity, and the macronutrient differences in comparison to OP40 were not significant in terms of the total protein, fat, and dietary fibre. Both were high-fibre (around 47%) and high-fat (approximately 17%) ingredients, and they also presented considerable amounts of protein (6.6%). Moreover, there was a slight but significant reduction in the ash content (OP40: 6.5% vs. OP70: 5.7% DW) and, consequently, a significant increase in the carbohydrate contents (OP70: 25% vs. OP40: 23% DW). OP70 showed higher values of antioxidants than OP40 (Table 2), in particular, in the total amounts of vitamin E (OP70: 86 vs. OP40: 73 mg/kg DW), phenolics (OP70: 3.4 vs. OP40: 2.7 g GAEs/100 g DW), and flavonoids (OP70: 3.5 vs. OP40: 2.3 g CEs/100 g DW), resulting in a higher antioxidant capacity in the FRAP assay (OP70: 7.5 vs. OP40: 4.8 g FSEs/100 g DW). However, that did not happen in the DPPH^●^-SA assay, in which the results were similar for both samples (>1 g TEs/100 g DW). Both samples also did not show differences in the overall FA profile, with both presenting mostly oleic acid (75–76%), but in a slightly higher content in the OP40 in relation to OP70 (12.7 vs. 12.4 g/100 g DW, respectively).

Nunes et al. (2018) obtained higher protein (around 7% vs. 4%) but lower fat (6% vs. 9%) and ash (2% vs. 4%) results for OP [6]. Nunes et al. (2020) also obtained a higher protein result for OP from Koroneiki cultivars (10% vs. 4%), but similar results for fat (9.7% vs. 9.2%) and ash (4.5% vs. 4.0%) [8]. Sousa et al. (2023) found lower levels of fat (3.6% vs. 9.23%), ash (2.7% vs. 4.03%), and fibre (44.0% vs. 54.68%), but higher levels of protein (6.3% vs. 4.25%) and carbohydrates (43.4% vs. 27.81%) for OP [10]. Similar to our study, the authors removed the stone by sieving, obtaining a paste-type product. In that paste, the authors found lower levels of fat (9.3% vs. 16.58–16.69% DW) and ash (4.9% vs. 5.65–6.46% DW) but higher fibre (48.0% vs. 42.42–42.66% DW), protein (9.6% vs. 6.59–6.61% DW), and carbohydrate (28.2% vs. 22.93–25.09% DW) contents compared to OP40 and OP70. Ferreira et al. (2024) reported a higher protein content (5.75%) and a slightly lower fat content (8.43%) in comparison to the present study [4]. Different edaphoclimatic conditions and olive varieties can justify the different compositions mentioned above [2].

Total vitamin E was significantly increased in the dried samples (OP70: 86 mg/kg > OP40: 73 mg/kg > FOP: 51 mg/kg DW), which once again can be explained by the stone removal. The increase in the total vitamin E with drying was also previously verified by Pasten et al. (2019) from almost 20.5 mg/100 g (at 40 °C) to 26.1 mg/100 g (at 70 °C) [44]. In the present study, α-tocopherol was also the main vitamer in all of the samples (OP70: 71 mg/kg > OP40: 59 mg/kg > FOP: 43 mg/kg). OP70 showed significantly higher results for all isomers in comparison to the FOP. The results of α-tocopherol, β-tocopherol, and γ-tocotrienol were also significantly higher in OP70 in contrast to OP40 (71 vs. 59 mg/kg, 2.5 vs. 2.2 mg/kg, and 6.6 vs. 6.1 mg/kg, respectively). These findings can be attributed to greater cell rupture during the sieving process, which likely allowed for the release of more vitamin E from inside of the cell organelles [10,44].

Nunes et al. (2018) presented higher results for α-tocopherol (76 vs. 42 mg/kg) but similar results for β-tocopherol (1 vs. 1 mg/kg) and γ-tocopherol (1 vs. 2 mg/kg) for OP [6]. Unlike the present study, α-tocotrienol was identified instead of γ-tocotrienol, δ-tocopherol, and δ-tocotrienol. Nunes et al. (2020) also identified α-tocotrienol but did not identify γ-tocotrienol, δ-tocopherol, and δ-tocotrienol [8]. The results were expressed in mg/g of oil, so it is not possible to directly compare the quantities, but rather only the identification of vitamers, with the present data.

Sousa et al. (2023) identified α-tocopherol, α-tocotrienol, β-tocopherol, γ-tocopherol, and δ-tocopherol, but did not identify γ-tocotrienol and δ-tocotrienol. The α-tocopherol content was similar (43.60 vs. 42.46 mg/kg). However, it totalised less vitamin E (43.60 vs. 51.30 mg/kg) compared to our results. Similar to our data, sieving increased the total vitamin E content from 43.60 to 61.01 mg/kg due to the stone removal [10]. Ferreira et al. (2024) only reported α-, β-, and γ-tocopherols, totalising an amount that is within our data if we consider the s.d. (48.6 vs. 51.30 ± 3.26 mg/kg) [4].

There were no significant differences in the FA profiles of the OP samples—the major FA was oleic acid (C18:1n9c, 75–76%), consequently traducing into great MUFA contents (77%). Nunes et al. (2018) obtained very similar percentages but, unlike this study, identified C14:0 (myristic acid), C17:0 (heptadecanoic acid), C17:1c (cis-10-heptadecenoic acid), C22:0 (behenic acid), and C20:1n9c (cis-11-eicosenoic acid) in OP. Nevertheless, these FAs were detected in very low percentages (<0.3%) [6]. Unlike the present data, Nunes et al. (2020) and Sousa et al. (2023) also identified C14:0, C17:0, C22:0, and C20:1n9c in OP [8,10]. The first authors [8] reported C18:1n9c percentages varying between 70.38% and 75.25%, whereas the latter [10] obtained the following values: 73.07% and 74.69%. Ferreira et al. (2024) determined values of around 75% and, similar to the present study, it did not identify C14:0 [4].

Pasten et al. (2019) also dried OP at 40 and 70 °C but, differing from the present work, used a lab-scale convective hot-air dryer, and their results revealed a significant increase in ash but a decrease in fat and protein contents with the increase in temperature [44]. In the TPC, TFC, FRAP, and DPPH^•^-SA assays, their results followed the same pattern in all of the assays—undried OP presented higher results than OP dried at 70 °C, followed by OP dried at 40 °C. In our study, the assays that followed that pattern were the TPC (3.9 > 3.4 > 2.7 g GAEs/100 g, respectively) and DPPH^•^-SA (1.5 ≈ 1.3 > 1.2 g TEs/100 g, respectively). On the contrary, OP70 showed significantly higher TFC (3.5 > 3.0 > 2.3 g CEs/100 g) and FRAP (7.5 > 5.4 ≈ 4.8 g FSEs/100 g) results than FOP and OP40, respectively. Pasten et al. (2019) used different unities in the antioxidant potential assays, impairing direct comparison, but obtained lower results for the TPC (around 2.6 > 1.6 > 1.3 g GAEs/100 g DW) and TFC (approximately 0.87 > 0.64 > 0.47 g CEs/100 g DW) assays in comparison to the present data [44]. Sousa et al. (2023) found slightly lower results for TPCs (3.08 g GAEs/100 g), TFC (2.69 g CEs/100 g), and FRAP (4.43 g FSEs/100 g), but a similar value in DPPH^•^-SA (1.53 g TEs/100 g) in relation to the present work [10]. Ferreira et al. (2024) reported similar TPCs (4.08 g GAEs/100 g) but a higher DPPH^•^-SA (4.28 g TEs/100 g) [4].

All in all, OP70 was chosen to be incorporated into the pasta because it not only dried faster, saving electricity, but also showed higher antioxidant values, particularly in total vitamin E, phenolics, and flavonoids, resulting in greater antioxidant capacity in the FRAP assay when compared to OP40. Additionally, the macronutrient differences between OP70 and OP40 were not significant, with both being high in fibre and fat and containing considerable amounts of protein. Despite a reduction in the ash content and a slight increase in carbohydrates, the superior antioxidant properties of OP70 made it the preferred choice for pasta enrichment.

### 3.2. Chemical Analysis of Pasta Samples

Pasta is traditionally made from semolina, a coarse yellow flour derived from durum wheat, known for its high gluten content that provides the desired texture, elasticity, and firmness. Semolina’s coarser texture and higher protein content enable the pasta to retain its shape and texture during cooking. In contrast, regular flour, with its finer texture and lower gluten content, can lead to a less firm and more easily overcooked pasta. This substitution may also affect cooking times and water absorption. Commercially, regular flour might reduce the production costs but could impact consumer acceptance and product quality, as semolina-based pasta is well-regarded for its texture [45,46,47].

**Figure 1:**
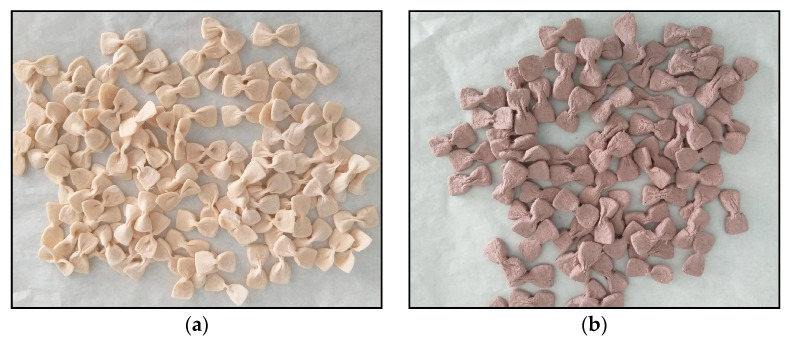
(**a**) Control pasta; (**b**) Olive pomace dried at 70 °C pasta.

Formulations 1 and 2 were manufactured without OP70, using wheat semolina or flour, respectively (Table 1). Both had a uniform texture after cooking, but formulation 2 (Figure 1a) was chosen as the control pasta because the enriched pasta chosen for analysis was made with wheat flour. Formulations 3 and 5, manufactured with OP70 and wheat semolina (Table 1), were rejected due to the granular texture after cooking. Formulation 4, containing 10% OP70 (Table 1), presented a granular texture after cooking, also being rejected. Formulation 6, produced with 7.5% OP70 (Table 1), had a uniform texture after cooking and was chosen for the analysis (Figure 1b).

The difference in texture observed between the semolina-based and flour-based formulations highlights the distinct roles of these ingredients when mixed with OP70. Regular flour, with its finer texture and lower gluten content, likely mixed better with OP70 than semolina, leading to textural changes. Semolina, with its coarser texture, likely mixed less effectively with OP70, producing a less cohesive formulation and resulting in a granular texture after cooking that led to the rejection of formulations 3 and 5. The quantity of OP70 also seems to impact the cohesiveness of the formulations, since, at 10% OP70, the formulations were rejected with both wheat products. While using flour may reduce production costs, it can also present challenges in maintaining the traditional texture associated with semolina pasta [45,46,47].

Table 3 presents the results of the chemical analysis of the pasta samples in DW. Both the uncooked control pasta (UCP) and cooked control pasta (CCP) exhibited the highest carbohydrate results (about 86%). Regarding the lipid fraction, the total vitamin E was significantly higher in the CCP compared to the UCP (10 mg/kg vs. 7 mg/kg), mostly β-tocotrienol (8 mg/kg vs. 5 mg/kg), which also happened for the major FA, linoleic acid (63% vs. 60%), totalising more PUFAs (66% vs. 63%, respectively). These samples showed very low results for total phenolics and flavonoids, as well as low levels in the FRAP assay. In fact, they were not detected in the DPPH^•^-SA assay.

The incorporation of 7.5% of OP70 into the pasta—uncooked olive pomace dried at 70 °C pasta (UOP70P)—increased the contents of fat (around 2% vs. 1% DW), ash (1.0% vs. 0.6% DW), total fibre (6% vs. 3% DW), soluble and insoluble fibre (3% vs. 2% and 3% vs. 1% DW, respectively), and vitamin E (27 vs. 7 mg/kg DW), but decreased the contents of protein (9% vs. 10% DW) and carbohydrates (82% vs. 86% DW) in relation to the UCP. There was also an increase in the energy value from 928 to 989 kJ/100 g. In kilocalories, the results were similar (395–397 kcal/100 g).

OP70 incorporation in the pasta not only allowed for the identification of γ-tocotrienol (1 mg/kg DW), but it also resulted in a significant increase for all isomers. After cooking, when comparing the UOP70P with the cooked olive pomace dried at 70 °C pasta (COP70P), there was a significant decrease in ash (1.0% vs. 0.8% DW) but an increase in protein (9% vs. 10% DW), whereas the other values continued similarly (Table 3).

α-Tocotrienol and β-tocotrienol were not identified in the by-product but were present in the pasta products, so it appears that they are probably provided by the wheat flour. Their amounts increase with by-product incorporation, so it seems possible that the pasta-making processing allowed for the release of more vitamin E, probably due to the higher rupture of some intact cells while mixing the ingredients and handling the dough, since the different isomers of vitamin E are located in different parts of plant cells, e.g., α-tocopherol is inside of the chloroplasts, while β-e γ-tocopherols are outside of the organelles [44].

OP70 incorporation in the pasta resulted in changes in the FA profiles—the major FAs shifted from linoleic acid in the control pasta (UCP: 60%, 475 mg/100 g vs. CCP: 63%, 444 mg/100 g) to oleic acid in the enriched pasta (UOP70P: 53%, 1121 mg/100 g vs. COP70P: 51%, 1061 mg/100 g). After cooking, it was not possible to identify C16:1*c*, C20:0, C20:1n9*c*, and C22:0, which is probably the result of fat transfer to the boiling water; nevertheless, they were present in low percentages (0.17–0.40%).

In another study, Padalino et al. (2018) identified C14:0 but did not identify C20:0, C20:1n9*c*, and C22:0 in pasta enriched with OP paste [16]. The major FA in their study was also oleic acid, but in lower amounts (39%). Durante et al. (2019) obtained similar results (the major FA was also oleic acid at 67%) in “Taralli” (a typical Italian bakery product) enriched with OP paste, but C17:0 was identified, unlike in the present study [18]. These differences can be explained by different ingredients and quantities used in the foodstuff manufacture.

COP70P (369 mg/100 g) and UOP70P (353 mg/100 g) had higher SFA levels than the UCP (169 mg/100 g) and CCP (144 mg/100 g). The MUFA sum showed the highest quantity in the UOP70P (1135 mg/100 g), significantly higher than the COP70P (1061 mg/100 g), while the UCP (119 mg/100 g) and CCP (92 mg/100 g) had much lower values. The PUFA sum was the highest in the COP70P (670 mg/100 g), then the UOP70P (612 mg/100 g), both of which were higher than the UCP (499 mg/100 g) and CCP (466 mg/100 g) (Table 3).

The OP70 pasta samples showed significantly higher results in the TPC, TFC, FRAP, and DPPH^•^-SA assays in comparison to the control pasta samples. Some of these compounds (phenolics and flavonoids) were probably lost or decomposed during cooking, since these parameters were reduced in the cooked products. In future work, it would be interesting to analyse the cooking water to understand if these compounds were extracted.

Simonato et al. (2019) also observed a decrease in TPCs and DPPH^•^-SA after cooking pasta fortified with OP. The cooked pasta fortified with 5% OP had 6.3-fold higher TPCs, 1.9-fold higher DPPH^●^-SA, and 23-fold higher ABTS than the cooked control. Similarly, the cooked pasta fortified with 10% OP resulted in even higher increases in these parameters (TPCs: 11.5-fold, DPPH^●^-SA: 4.8-fold, and ABTS: 77-fold higher) than the cooked control. In both percentages, pasta fortification resulted in a decrease in the rapidly digestible starch content. Instead, there was an increase in the slowly digestible and resistant starch [19].

Table 4 presents the chemical analysis of the cooked pasta samples in FW, reflecting how this foodstuff is typically consumed. OP70 incorporation in pasta led to a significant decrease in the water absorbed during cooking (CCP: 62% vs. COP70P: 43% FW); consequently, COP70P presented the highest macronutrient results (carbohydrates: 47%, protein: 5.8%, fat: 1.2%, ash: 0.4%, and fibre: 3.1% FW) and energy value (228 kcal/100 g). Enriched pasta showed significantly higher contents for all vitamin E isomers, total phenolics and flavonoids, as well as the FRAP assay. Notably, COP70P was the only sample capable of scavenging DPPH^•^ (Table 4) and containing γ-tocotrienol (0.6 mg/kg FW).

Vitamin E is indispensable in human nutrition and is only obtained from the diet [48]. Its deficiency can cause ataxia and an enhanced risk of developing conditions related to fat malabsorption, so it is essential for a healthy life. The daily consumption levels of α-tocopherol are 13 mg and 11 mg for adult males and females, respectively [49]. The amount of α-tocopherol found in COP70P (about 4.2 mg/kg FW) might help to contribute to its daily consumption, since it is the most biologically active isomer in humans [50].

Even though the total fat content increased in COP70P in comparison to the control (1.2% vs. 0.3% FW), presenting a higher energetic contribution, the fat was composed mostly of oleic acid, which is a partially essential FA because the body is not able to fully compensate its synthesis in diets low in this compound [51]. So, the consumption of the enriched pasta could contribute in this way. FAs are also crucial to the brain and the peripheral nervous system as an energy source, molecule signalling agents, and as building blocks of cellular membranes [51].

Oleic acid is associated with the following three key concepts of metabolic syndrome: obesity, insulin resistance, and type 2 diabetes mellitus [51]. A reduction in SFA contents and higher MUFA contents in diets are associated with improved insulin sensitivity, protection against certain types of cancer, and a lower risk of developing cardiovascular and neurodegenerative diseases [50,51,52,53]. Additionally, OP70 incorporation in the pasta increased the MUFA (>50%) and decreased the PUFA contents (Table 3), which, in combination with the vitamin E levels, can offer greater resistance to oxidation and might contribute to longer pasta shelf-life [50].

A healthy status is correlated with a 1:1 intake proportion of n6-PUFA to n3-PUFA, preventing negative effects such as neuroinflammation and loss of memory associated with Alzheimer’s and Parkinson’s diseases. The WHO recommends an even higher ratio of 5:1 to 10:1, similar to that found in olive oil [51]. Our results were higher than the recommended values (n6/n3: almost 18), which can also be an advantage of consuming this enriched pasta. The recommended nutritional guidelines for PUFA/SFA ratios are >four-tenths [51]. Our findings revealed that the incorporation of OP70 in pasta was beneficial in this parameter, with a shift from three-tenths in the control to almost six-tenths in the OPP.

Based on all of the previous results, we can conclude that incorporating OP70 improved the nutritional composition of the pasta, presenting potential health benefits.

In future research, it would be interesting to determine the minerals, amino acids, and sugars of the final pasta samples of our study.

Additionally, in the developed formulation, OP partially substitutes the wheat flour. Scaling up production in the food industry could have a beneficial impact due to the large amount of OP available each year from olive oil production. This is particularly relevant in Portugal, where around 600,000 tons of this agri-food waste was produced in 2021 [54], creating several struggles for olive oil producers and environmental concerns.

### 3.3. Sensory Analysis of Pasta Samples

The acceptability test and consumer survey were helpful to identify the specific sensory attributes of the pasta samples that are relevant to predict the acceptance of the products. The preference test confirmed the previous results as well as the buying intention, which is highly correlated with acceptability.

The demographic characterisation of the consumer panel (*n* = 71) had the following age distribution: ≤18 (5 participants), 19–30 (15 participants), 31–49 (29 participants), and 50–69 (22 participants); no one was 70 years old or older. Regarding the frequency of pasta consumption, most participants consumed pasta “Frequently” (52 participants), “Occasionally” (11 participants), “Very frequently” (6 participants), and “Rarely” (2 participants). No one answered “Never”.

**Figure 2:**
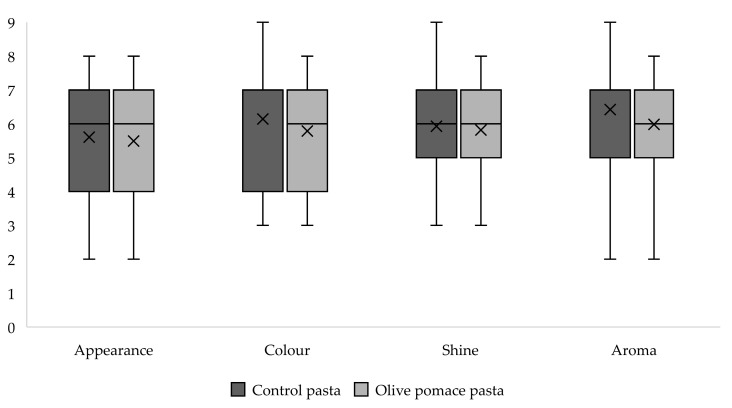
Boxplot of acceptability scores before tasting.

Figure 2 shows the scores attributed before tasting the pasta samples. Very similar results were obtained for parameters such as appearance, colour, shine, and aroma for the control pasta (CP) and olive pomace pasta (OPP). More than half of the consumers rated both formulations positively in terms of appearance, colour, shine, and aroma (with scores from six to nine points on the hedonic scale, corresponding to “Slightly pleasant”, “Pleasant”, “Very pleasant”, and “Extremely pleasant” opinions, respectively). This highlights the potential acceptability of the new product. Regarding appearance, the preferred sample was the CP, which was considered “Slightly pleasant” or higher by 56% of the consumers, slightly more than the OPP (54%). Concerning colour, the CP was more appreciated (69% vs. 58% gave a positive score). Most individuals also liked the products’ brightness (shine), in this case, preferably the enriched pasta (66% vs. 59% gave a positive score). The majority of participants (72%) also considered the aroma of both samples pleasant.

**Figure 3:**
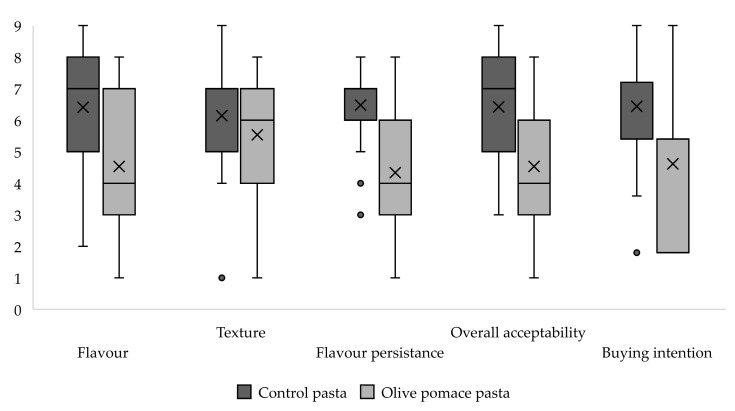
Boxplot of acceptability scores after tasting (buying intention results were multiplied by a 9/5 factor, allowing us to plot these results in the same graph).

Figure 3 shows the scores attributed after tasting the pasta samples. Concerning the pasta texture, the CP revealed a slightly higher acceptability than the OPP. This difference is more noticeable in flavour, flavour persistence, overall acceptability, and buying intention. The preferred pasta after tasting, considering flavour, was the control (72% gave a positive score), whereas the OPP was rejected by 52% of the tasters. The OPP was even classified as “Extremely unpleasant” by nine consumers. Nevertheless, the majority of the pasta consumers liked the texture of both pasta samples (CP: 68% and OPP: 59%), but the control was the only one classified as “Extremely pleasant” by two participants. Regrettably, the flavour persistence of the enriched pasta was not appreciated—it received a negative score (from one to four points, corresponding to “Slightly unpleasant”, “Unpleasant”, “Very unpleasant”, and “Extremely unpleasant” opinions, respectively) by 55% of the inquired population, and three consumers even classified it as “Extremely unpleasant”.

The less favourable reception of the OPP in terms of flavour and flavour persistence may be linked to the volatile compounds present in OP. While we have not yet conducted a specific analysis of these compounds in our study, the literature suggests that certain volatile compounds in OP could contribute to these sensory perceptions. Styrene, a derivative of benzene, may impart distinct aromatic qualities that are not universally appealing. FAs, such as 2-propenoic acid, hexadecanoic acid, and 9-octadecenoic acid, are known to influence flavour profiles significantly, potentially contributing to the lingering, less desirable taste. Additionally, 2-hexadecanol, a fatty alcohol, might further affect the overall aroma and taste, leading to the observed rejection or lower appreciation of the enriched pasta compared to the control. Understanding these compounds could provide valuable insights into improving the flavour profile of OP-enriched pasta in future formulations [55].

The overall acceptability revealed that consumers preferred the CP—75% of them attributed a positive score (above six) to it. Less than half liked the OPP (39%), with 55% giving it a negative score (less than four), and a taster even classified it as “Extremely unpleasant”, while the CP was classified as “Extremely pleasant” by two consumers.

**Figure 4:**
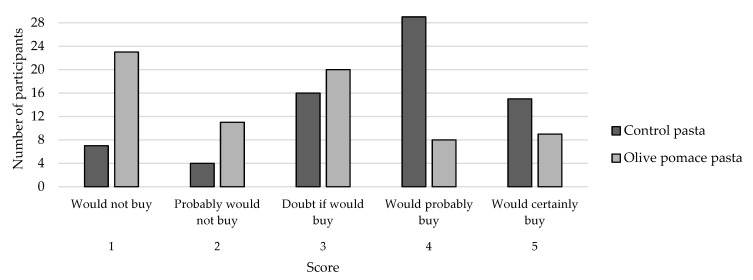
Buying intention of pasta.

Considering the buying intention (Figure 4), the CP was the one with a better chance of being bought (62% would buy). On the contrary, 48% would not buy the OPP. It is important to point out that the price factor was not included in our analysis, as this pasta is still in the prototype stage. We plan to conduct economic and feasibility studies for the new pasta in the future, including a thorough assessment of the production costs, taking into account factors such as large-scale production, labour costs, energy and water usage, and packaging. However, we anticipate that, by substituting part of the wheat with a by-product that is considered a waste, food manufacturers may be able to slightly reduce the price of the enriched pasta sample.

Regarding the preference test, 76% of the consumers preferred the CCP over the COPP, probably because it is the most like the majority of pasta products available in the market.

In a previous study, the incorporation of 10% dried OP flour in fish burgers resulted in higher TPCs and TFC (12.8-fold and 210.2-fold, respectively) after cooking in comparison to the control, but, similarly to our study, it also had a negative impact on the sensory attributes due to their very bitter and spicy taste [30].

Panza et al. (2020) used a lower amount of OP powder (0.2%) to coat cod sticks; consequently, the sensory acceptability of the cooked sticks was not compromised. After cooking, the OP-breaded sticks showed a 4.4-fold higher TPC, 7.7-fold higher TFC, and a 2.9-fold higher antioxidant activity compared to the control. Microbiological quality was maintained for more time in the cooked sticks with the OP than in the control [31].

In another work, 15% of OP powder was used to manufacture biscuits; the incorporation improved the nutritional properties (fibre increased by 9-fold). It resulted in higher scores in the sensory evaluation (colour, appearance, taste, texture, and overall acceptability) than the control biscuits [22].

Different bakery goods were also previously formulated, namely, with a granola bar fortified with 5% OP, which showed high hydroxytyrosol and tyrosol contents of 36.4 and 6.5 mg/100 g, respectively. Colour and bitter taste were also negatively affected by the fortification [17].

The present sensory analysis results suggest that a lower percentage of the by-product should be hypothesised in future formulations, e.g., 6% or 5%. Pasta manufacturing should also be attempted using industrial machines. So far, production has only taken place in the lab with limited resources and without the proper technology. Producing pasta products more similar to those in the food market would certainly improve consumer acceptability. Hence, after the optimisation of the formulation, this prototype pasta has strong potential for mass production in the food industry with the proper technology and equipment.

Nevertheless, even if the developed pasta is not a product accepted by the majority of consumers, it can be restricted to a niche market, e.g., for people who follow sustainable diets. Moreover, this new product is suitable for plant-based food patterns. Indeed, the Portuguese food market has similar pasta products containing peas, chickpeas, lentils, oats, spelt, quinoa, teff, buckwheat, amaranth, among others [56,57]. We have carried out sensory tasting tests of some of those products in the lab and concluded that our final pasta was similar in terms of texture, resembling whole-grain products, and, in some cases, flavour persistence. Moreover, algae pasta [58] is also commercially available [56,57], which can resemble our product in colour. Therefore, our prototype pasta can be an alternative to other pasta options enriched with vegetables and cereals currently available in the food market.

In future work, we intend to focus on the following case study: how to mask the organoleptic effects of adding OP to pasta while retaining its nutritional benefits. Several corrective actions can be hypothesised and implemented, such as the optimisation of the OP proportions to find a balance that maintains the nutritional benefits while improving the sensory qualities; flavour masking by incorporating additional flavourings or seasonings (e.g., herbs and spices) to mask any undesirable tastes or to enhance the overall flavour profile of the enriched pasta; textural adjustments (modifying the pasta-making process or recipe to improve texture, ensuring that the enriched pasta has a more appealing mouthfeel); consumer feedback integration to gather detailed feedback and refine the product accordingly. All of these could enhance consumer acceptance while still benefiting from the nutritional advantages of the enriched pasta.

## 4. Conclusions

FOP is a good source of dietary fibre, mostly insoluble, and fat composed mainly by oleic acid and α-tocopherol. The applied drying and sieving procedures caused increases in protein, fat, ash, soluble fibre, and vitamin E, but decreases in total and insoluble fibre and carbohydrate contents due to the stone removal, without affecting the FA profiles. While the FOP had the highest results in TPCs and DPPH^•^-SA, OP70 excelled in TFC and FRAP.

A 7.5% OP70 incorporation in the pasta resulted in higher contents of fat, ash, fibre, vitamin E, oleic acid, and MUFAs relative to the control. The analysed antioxidants (vitamin E, TPCs, and TFC) were higher in the enriched pasta compared to the control but partially lost during cooking. In contrast, the overall nutritional composition was improved in terms of macronutrients and FAs. The sensory analysis revealed that consumers appreciated the appearance, colour, shine, and aroma of both the control and enriched pasta samples. However, after tasting, parameters like flavour and texture were impaired by the OP70 incorporation, compromising the overall acceptability and buying intention.

Nevertheless, this study shows that this by-product can be used to develop a sustainable pasta with enhanced nutritional benefits, supporting circular economy principles and food security. It offers an alternative for plant-based diets and current pasta options.

## Figures and Tables

**Table 1:** Composition of pasta formulations (OP70—olive pomace dried at 70 °C).

Formulation	OP70 %	OP70 (g)	Wheat Semolina (g)	Wheat Flour (g)	Water (mL)
1	-	-	200	-	110
2	-	-	-	200	110
3	10%	20	180	-	110
4	10%	20	-	180	110
5	7.5%	15	185	-	110
6	7.5%	15	-	185	110

**Table 2:** Chemical analysis of freeze-dried olive pomace (FOP), olive pomace dried at 40 °C (OP40), and olive pomace dried at 70 °C (OP70).

Sample	FOP	OP40	OP70	*p*-Value
Fat (g/100 g)	9.23 ± 0.26 ^b^	16.69 ± 0.14 ^a^	16.58 ± 0.19 ^a^	***
Ash (g/100 g)	4.03 ± 0.07 ^c^	6.46 ± 0.07 ^a^	5.65 ± 0.10 ^b^	***
Dietary fibre (g/100 g)	54.68 ± 0.54 ^a^	47.31 ± 0.27 ^b^	47.23 ± 0.05 ^b^	***
Insoluble fibre (g/100 g)	51.43 ± 0.31 ^a^	42.66 ± 0.33 ^b^	42.42 ± 0.53 ^b^	***
Soluble fibre (g/100 g)	3.25 ± 0.23	4.65 ± 0.06	4.80 ± 0.48	
Protein (g/100 g)	4.25 ± 0.09 ^b^	6.61 ± 0.09 ^a^	6.59 ± 0.39 ^a^	***
Carbohydrates (g/100 g)	27.81 ± 0.22 ^a^	22.93 ± 0.16 ^c^	25.09 ± 0.26 ^b^	***
α-Tocopherol (mg/kg)	42.46 ± 2.94 ^c^	58.87 ± 1.04 ^b^	70.83 ± 1.07 ^a^	***
β-Tocopherol (mg/kg)	1.36 ± 0.03 ^c^	2.19 ± 0.02 ^b^	2.48 ± 0.08 ^a^	***
γ-Tocopherol (mg/kg)	2.48 ± 0.09 ^b^	3.90 ± 0.02 ^a^	4.06 ± 0.05 ^a^	***
γ-Tocotrienol (mg/kg)	3.71 ± 0.21 ^c^	6.12 ± 0.08 ^b^	6.63 ± 0.08 ^a^	***
δ-Tocopherol (mg/kg)	1.29 ± 0.01 ^b^	2.25 ± 0.04 ^a^	2.34 ± 0.04 ^a^	***
Total vitamin E (mg/kg)	51.30 ± 3.26 ^c^	73.33 ± 1.18 ^b^	86.34 ± 1.21 ^a^	***
C16:0 Palmitic acid (relative %)	10.40 ± 0.06	10.59 ± 0.11	10.47 ± 0.15	
C16:1*c* Palmitoleic acid (relative %)	0.48 ± 0.04	0.54 ± 0.05	0.46 ± 0.06	
C18:0 Stearic acid (relative %)	3.38 ± 0.19	3.36 ± 0.24	3.65 ± 0.08	
C18:1n9*c* Oleic acid (relative %)	76.19 ± 0.24	76.28 ± 0.89	75.04 ± 0.09	
C18:2n6*c* Linoleic acid (relative %)	8.70 ± 0.49	8.38 ± 0.69	9.44 ± 0.12	
C20:0 Arachidic acid (relative %)	0.31 ± 0.02	0.27 ± 0.04	0.32 ± 0.03	
C18:3n3*c* α-Linolenic acid (relative %)	0.45 ± 0.07	0.49 ± 0.07	0.53 ± 0.02	
C24:0 Lignoceric acid (relative %)	0.07 ± 0.02	0.08 ± 0.01	0.09 ± 0.01	
∑SFAs Saturated fatty acids (relative %)	14.17 ± 0.19	14.30 ± 0.14	14.52 ± 0.10	
∑MUFAs Monounsaturated fatty acids (relative %)	76.67 ± 0.27 ^a, b^	76.82 ± 0.85 ^a^	75.50 ± 0.03 ^b^	***
∑PUFAs Polyunsaturated fatty acids (relative %)	9.16 ± 0.46	8.88 ± 0.75	9.97 ± 0.11	
C16:0 Palmitic acid (g/100 g)	0.96 ± 0.00 ^b^	1.77 ± 0.02 ^a^	1.74 ± 0.02 ^a^	***
C16:1*c* Palmitoleic acid (g/100 g)	0.04 ± 0.00 ^b^	0.09 ± 0.01 ^a^	0.08 ± 0.01 ^a^	***
C18:0 Stearic acid (g/100 g)	0.31 ± 0.01 ^b^	0.56 ± 0.03 ^a^	0.60 ± 0.01 ^a^	***
C18:1n9*c* Oleic acid (g/100 g)	7.03 ± 0.02 ^c^	12.73 ± 0.12 ^a^	12.44 ± 0.01 ^b^	***
C18:2n6*c* Linoleic acid (g/100 g)	0.80 ± 0.04 ^b^	1.40 ± 0.09 ^a^	1.57 ± 0.02 ^a^	***
C20:0 Arachidic acid (g/100 g)	0.03 ± 0.00 ^b^	0.04 ± 0.00 ^a^	0.05 ± 0.00 ^a^	*
C18:3n3*c* α-Linolenic acid (g/100 g)	0.04 ± 0.01 ^b^	0.08 ± 0.01 ^a^	0.09 ± 0.00 ^a^	**
C24:0 Lignoceric acid (g/100 g)	0.01 ± 0.00 ^b^	0.01 ± 0.00 ^a^	0.02 ± 0.00 ^a^	**
∑SFAs Saturated fatty acids (g/100 g)	1.31 ± 0.01 ^b^	2.39 ± 0.02 ^a^	2.41 ± 0.02 ^a^	**
∑MUFAs Monounsaturated fatty acids (g/100 g)	7.08 ± 0.02 ^c^	12.82 ± 0.12 ^a^	12.52 ± 0.01 ^b^	**
∑PUFAs Polyunsaturated fatty acids (g/100 g)	0.85 ± 0.04 ^b^	1.48 ± 0.10 ^a^	1.65 ± 0.02 ^a^	***
n6/n3	19.56 ± 3.03	17.10 ± 1.16	17.78 ± 0.52	
PUFAs/SFAs	0.65 ± 0.03	0.62 ± 0.04	0.69 ± 0.01	
TPCs (g GAEs/100 g)	3.92 ± 0.49 ^a^	2.73 ± 0.15 ^c^	3.38 ± 0.15 ^b^	**
TFC (g CEs/100 g)	3.00 ± 0.39 ^b^	2.26 ± 0.13 ^c^	3.46 ± 0.11 ^a^	**
FRAP (g FSEs/100 g)	5.37 ± 0.95 ^b^	4.84 ± 0.34 ^b^	7.49 ± 0.33 ^a^	***
DPPH^•^-SA (g TEs/100 g)	1.50 ± 0.23 ^a^	1.18 ± 0.09 ^b^	1.27 ± 0.12 ^a,b^	*

Results are expressed as mean ± standard deviation (*n* = 3) in dry weight. In each line, different superscript letters represent significant differences between samples (* *p* ≤ 0.05, ** *p* ≤ 0.01, and *** *p* ≤ 0.001). TPCs—total phenolic compounds, GAEs—gallic acid equivalents, TFC—total flavonoid content, CEs—catechin equivalents, FRAP—ferric reducing antioxidant power, FSEs—ferrous sulphate equivalents, DPPH^•^-SA—2,2-diphenyl-1-picrylhydrazyl radical scavenging ability, TEs—Trolox equivalents.

**Table 3:** Chemical analysis of uncooked control pasta (UCP), cooked control pasta (CCP), uncooked olive pomace dried at 70 °C pasta (UOP70P), and cooked olive pomace dried at 70 °C pasta (COP70P).

Sample	UCP	CCP	UOP70P	COP70P	*p*-Value
Energy (kJ/100 g)	928 ± 5 ^b^	933 ± 3 ^b^	989 ± 3 ^a^	995 ± 4 ^a^	***
Energy (kcal/100 g)	397 ± 1 ^a,b^	397 ± 0 ^a^	395 ± 0 ^b^	397 ± 0 ^a^	*
Fat (g/100 g)	0.79 ± 0.13 ^b^	0.70 ± 0.02 ^b^	2.10 ± 0.05 ^a^	2.10 ± 0.05 ^a^	***
Ash (g/100 g)	0.58 ± 0.01 ^c^	0.44 ± 0.02 ^d^	0.96 ± 0.01 ^a^	0.75 ± 0.01 ^b^	***
Dietary fibre (g/100 g)	2.52 ± 0.12 ^b^	2.48 ± 0.18 ^b^	5.93 ± 0.04 ^a^	5.45 ± 0.43 ^a^	**
Insoluble fibre (g/100 g)	0.95 ± 0.03 ^b^	1.00 ± 0.02 ^b^	2.88 ± 0.10 ^a^	2.55 ± 0.36 ^a^	*
Soluble fibre (g/100 g)	1.58 ± 0.15 ^b^	1.48 ± 0.20 ^b^	3.05 ± 0.05 ^a^	2.90 ± 0.09 ^a^	**
Protein (g/100 g)	9.64 ± 0.02 ^b^	10.48 ± 0.15 ^a^	9.11 ± 0.19 ^c^	10.14 ± 0.13 ^a^	*
Carbohydrates (g/100 g)	86.46 ± 0.14 ^a^	85.89 ± 0.18 ^a^	81.90 ± 0.22 ^b^	81.56 ± 0.18 ^b^	***
α-Tocopherol (mg/kg)	0.58 ± 0.02 ^b^	0.69 ± 0.01 ^b^	7.38 ± 0.11 ^a^	7.32 ± 0.07 ^a^	***
α-Tocotrienol (mg/kg)	0.28 ± 0.00 ^b^	0.30 ± 0.00 ^b^	1.49 ± 0.08 ^a^	1.49 ± 0.02 ^a^	***
β-Tocopherol (mg/kg)	0.68 ± 0.02 ^c^	1.01 ± 0.01 ^b^	2.10 ± 0.04 ^a^	2.09 ± 0.02 ^a^	***
γ-Tocopherol (mg/kg)	0.47 ± 0.01 ^c^	0.60 ± 0.00 ^b^	0.91 ± 0.01 ^a^	0.91 ± 0.01 ^a^	***
β-Tocotrienol (mg/kg)	4.92 ± 0.17 ^c^	7.61 ± 0.04 ^b^	14.14 ± 0.33 ^a^	14.14 ± 0.19 ^a^	***
γ-Tocotrienol (mg/kg)	ND	ND	1.01 ± 0.09 ^a^	1.06 ± 0.09 ^a^	***
Total vitamin E (mg/kg)	6.93 ± 0.21 ^c^	10.21 ± 0.04 ^b^	27.03 ± 0.58 ^a^	27.00 ± 0.35 ^a^	***
C16:0 Palmitic acid (relative %)	19.93 ± 0.40 ^a^	19.39 ± 0.09 ^a^	13.63 ± 0.22 ^c^	14.85 ± 0.35 ^b^	**
C16:1*c* Palmitoleic acid (relative %)	ND	ND	0.40 ± 0.01	ND	
C18:0 Stearic acid (relative %)	1.54 ± 0.10 ^b^	1.16 ± 0.07 ^b^	2.78 ± 0.03 ^a^	2.70 ± 0.26 ^a^	***
C18:1n9*c* Oleic acid (relative %)	14.68 ± 0.49 ^c^	12.47 ± 0.46 ^d^	53.39 ± 0.40 ^a^	50.52 ± 0.23 ^b^	***
C18:2n6*c* Linoleic acid (relative %)	60.32 ± 0.46 ^b^	63.12 ± 0.70 ^a^	27.61 ± 0.22 ^d^	30.20 ± 0.51 ^c^	***
C20:0 Arachidic acid (relative %)	ND	ND	0.21 ± 0.02	ND	
C18:3n3*c* Linolenic acid (relative %)	3.12 ± 0.20 ^a^	3.20 ± 0.14 ^a^	1.55 ± 0.01 ^b^	1.72 ± 0.03 ^b^	***
C20:1n9*c cis*-11-eicosenoic acid (relative %)	0.41 ± 0.03 ^b^	0.67 ± 0.06 ^a^	0.25 ± 0.02 ^c^	ND	
C22:0 Behenic acid (relative %)	ND	ND	0.17 ± 0.01	ND	
∑SFAs Saturated fatty acids (relative %)	21.47 ± 0.49 ^a^	20.55 ± 0.08 ^b^	16.79 ± 0.20 ^c^	17.56 ± 0.42 ^c^	*
∑MUFAs Monounsaturated fatty acids (relative %)	15.09 ± 0.45 ^c^	13.13 ± 0.50 ^d^	54.04 ± 0.40 ^a^	50.52 ± 0.23 ^b^	**
∑PUFAs Polyunsaturated fatty acids (relative %)	63.44 ± 0.46 ^b^	66.32 ± 0.56 ^a^	29.16 ± 0.22 ^d^	31.92 ± 0.52 ^c^	***
C16:0 Palmitic acid (mg/100 g)	156.89 ± 2.54 ^c^	136.30 ± 0.49 ^d^	286.27 ± 3.76 ^b^	311.94 ± 6.09 ^a^	**
C16:1*c* Palmitoleic acid (mg/100 g)	ND	ND	8.46 ± 0.23	ND	
C18:0 Stearic acid (mg/100 g)	12.13 ± 0.62 ^b^	8.17 ± 0.42 ^b^	58.32 ± 0.44 ^a^	56.80 ± 4.52 ^a^	***
C18:1n9*c* Oleic acid (mg/100 g)	115.55 ± 3.13 ^c^	87.65 ± 2.65 ^d^	1121.33 ± 6.78 ^a^	1061.05 ± 3.92 ^b^	***
C18:2n6*c* Linoleic acid (mg/100 g)	474.83 ± 2.98 ^c^	443.82 ± 4.00 ^d^	579.93 ± 3.80 ^b^	634.20 ± 8.73 ^a^	**
C20:0 Arachidic acid (mg/100 g)	ND	ND	4.50 ± 0.31	ND	
C18:3n3*c* α-Linolenic acid (mg/100 g)	24.53 ± 1.26 ^c^	22.48 ± 0.80 ^c^	32.55 ± 0.12 ^b^	36.07 ± 0.58 ^a^	**
C20:1n9*c cis*-11-eicosenoic acid (mg/100 g)	3.24 ± 0.21 ^b^	4.69 ± 0.34 ^a^	5.22 ± 0.37 ^a^	ND	**
C22:0 Behenic acid (mg/100 g)	ND	ND	3.62 ± 0.22	ND	
∑SFAs Saturated fatty acids (mg/100 g)	169.02 ± 3.13 ^c^	144.47 ± 0.47 ^d^	352.71 ± 3.51 ^b^	368.74 ± 7.22 ^a^	*
∑MUFAs Monounsaturated fatty acids (mg/100 g)	118.79 ± 2.92 ^c^	92.33 ± 2.87 ^d^	1135.01 ± 6.86 ^a^	1061.05 ± 3.92 ^b^	**
∑PUFAs Polyunsaturated fatty acids (mg/100 g)	499.36 ± 2.93 ^c^	466.30 ± 3.20 ^d^	612.49 ± 3.80 ^b^	670.26 ± 8.96 ^a^	***
n6/n3	19.41 ± 1.03	19.77 ± 0.90	17.81 ± 0.14	17.59 ± 0.30	
PUFAs/SFAs	0.34 ± 0.01 ^b^	0.31 ± 0.00 ^b^	0.58 ± 0.00 ^a^	0.55 ± 0.02 ^a^	***
TPCs (g GAEs/100 g)	0.090 ± 0.008 ^c^	0.030 ± 0.002 ^d^	0.239 ± 0.013 ^a^	0.198 ± 0.010 ^b^	***
TFC (g CEs/100 g)	0.001 ± 0.001 ^c^	0.007 ± 0.000 ^c^	0.234 ± 0.018 ^a^	0.212 ± 0.017 ^b^	*
FRAP (g FSEs/100 g)	0.281 ± 0.013 ^c^	0.254 ± 0.010 ^c^	3.470 ± 0.187 ^a^	3.011 ± 0.133 ^b^	***
DPPH^•^-SA (g TEs/100 g)	ND	ND	0.102 ± 0.016	0.092 ± 0.021	

Results are expressed as mean ± standard deviation (*n* = 3) in dry weight. In each line, different superscript letters represent significant differences between samples (* *p* ≤ 0.05, ** *p* ≤ 0.01, and *** *p* ≤ 0.001). ND—not detected, TPCs—total phenolic compounds, GAEs—gallic acid equivalents, TFC—total flavonoid content, CEs—catechin equivalents, FRAP—ferric reducing antioxidant power, FSEs—ferrous sulphate equivalents, DPPH^•^-SA—2,2-diphenyl-1-picrylhydrazyl radical scavenging ability, TEs—Trolox equivalents.

**Table 4:** Chemical analysis of cooked control pasta (CCP) and cooked olive pomace dried at 70 °C pasta (COP70P).

Sample	CCP	COP70P	*p*-Value
Energy (kJ/100 g)	354 ± 1 ^b^	573 ± 2 ^a^	*
Energy (kcal/100 g)	150 ± 0 ^b^	228 ± 0 ^a^	*
Moisture (g/100 g)	62.1 ± 0.8 ^a^	42.5 ± 0.8 ^b^	*
Fat (g/100 g)	0.27 ± 0.01 ^b^	1.21 ± 0.03 ^a^	*
Ash (g/100 g)	0.17 ± 0.01 ^b^	0.43 ± 0.01 ^a^	*
Dietary fibre (g/100 g)	0.94 ± 0.07 ^b^	3.13 ± 0.25 ^a^	*
Insoluble fibre (g/100 g)	0.38 ± 0.01 ^b^	1.47 ± 0.20 ^a^	*
Soluble fibre (g/100 g)	0.56 ± 0.08 ^b^	1.67 ± 0.05 ^a^	*
Protein (g/100 g)	3.97 ± 0.06 ^b^	5.84 ± 0.07 ^a^	*
Carbohydrates (g/100 g)	32.56 ± 0.07 ^b^	46.93 ± 0.10 ^a^	*
α-Tocopherol (mg/kg)	0.26 ± 0.00 ^b^	4.21 ± 0.04 ^a^	*
α-Tocotrienol (mg/kg)	0.11 ± 0.00 ^b^	0.85 ± 0.01 ^a^	*
β-Tocopherol (mg/kg)	0.38 ± 0.00 ^b^	1.20 ± 0.01 ^a^	*
γ-Tocopherol (mg/kg)	0.23 ± 0.00 ^b^	0.52 ± 0.01 ^a^	*
β-Tocotrienol (mg/kg)	2.89 ± 0.01 ^b^	8.13 ± 0.11 ^a^	*
γ-Tocotrienol (mg/kg)	ND	0.61 ± 0.05	
Total vitamin E (mg/kg)	3.87 ± 0.01 ^b^	15.54 ± 0.20 ^a^	*
TPCs (g GAEs/100 g)	0.011 ± 0.001 ^b^	0.116 ± 0.005 ^a^	*
TFC (g CEs/100 g)	0.002 ± 0.002 ^b^	0.122 ± 0.008 ^a^	*
FRAP (g FSEs/100 g)	0.096 ± 0.004 ^b^	1.732 ± 0.076 ^a^	*
DPPH^•^-SA (g TEs/100 g)	ND	0.054 ± 0.011	

Results are expressed as mean ± standard deviation (*n* = 3) in fresh weight. In each line, different superscript letters represent significant differences between samples (* *p* ≤ 0.001). ND—not detected, TPCs—total phenolic compounds, GAEs—gallic acid equivalents, TFC—total flavonoid content, CEs—catechin equivalents, FRAP—ferric reducing antioxidant power, FSE—ferrous sulphate equivalents, DPPH^•^-SA—2,2-diphenyl-1-picrylhydrazyl radical scavenging ability, TEs—Trolox equivalents.

## Data Availability

The original contributions presented in the study are included in the article, further inquiries can be directed to the corresponding author.
